# Multifocal Pulmonary Opacities in an Elderly Smoker

**DOI:** 10.7759/cureus.73793

**Published:** 2024-11-16

**Authors:** Patton C McClelland, Zachary Jarrett, Christian C Lamb, Mateo Houle

**Affiliations:** 1 Internal Medicine, Brooke Army Medical Center, San Antonio, USA; 2 Pulmonary and Critical Care Medicine Service, Brooke Army Medical Center, San Antonio, USA

**Keywords:** histology, imaging, lung cancer, organizing pneumonia, pathology, pulmonary opacity, radiology, smoking, tobacco

## Abstract

Organizing pneumonia (OP) directly caused by ongoing cigarette smoking does not appear to have been previously described. Despite OP having pathophysiological features distinct from lung cancer, the two may be confused based on similar clinical, radiological, and histopathological findings. This distinction is further clouded by the dynamic nature of these diseases. Diagnostic accuracy can likely be improved with a multidisciplinary approach that involves input from several specialists and serial reevaluations as the disease evolves. We present a case of OP that was initially misdiagnosed as non-small-cell lung cancer and resolved with smoking cessation.

## Introduction

Inhaled tobacco smoke is a well-known cause of airway and lung disease, including chronic obstructive pulmonary disease (COPD) and malignancy [1]. Smoking has been linked to the development of interstitial lung diseases, such as smoking-related interstitial pneumonia (IIP), an umbrella term that includes desquamative interstitial pneumonia (DIP) and respiratory bronchiolitis-interstitial lung disease (RB-ILD) [1-3]. Smoking cigarettes has also been associated with pulmonary Langerhans cell histiocytosis, acute eosinophilic pneumonia, bronchiolitis, smoking-related interstitial fibrosis, combined pulmonary fibrosis and emphysema, and idiopathic pulmonary fibrosis [1-3]. With so many lung and airway diseases linked to cigarette use, a multimodal approach combining clinical, radiological, and pathological findings is useful when distinguishing between these entities and other rare conditions [2].

Organizing pneumonia (OP) is well described; however, smoking as a cause of OP is not. OP involves pulmonary tissue repair that can be idiopathic (i.e., cryptogenic) or due to drug-induced injury, inhalation of pathogens or toxic gas, gastroesophageal reflux disease, radiotherapy, organ transplant, or collagenosis [4]. It is histopathologically defined by the presence of granulation tissue consisting of fibroblasts-myofibroblasts rooted in connective tissue within intra-alveolar buds [5]. OP is reversible, may improve rapidly with corticosteroid use, and tends to relapse after cessation of treatment [5]. Manifestations of the idiopathic form of OP, termed cryptogenic organizing pneumonia (COP), include constitutional symptoms such as fever, malaise, weight loss, and anorexia, as well as cough and dyspnea. Hemoptysis is uncommon [5].

The presentation of OP can be similar to that of lung cancer. Imaging findings are variable and evolving and may imitate the appearance of other lung pathologies [4]. However, some distinguishing pulmonary findings include a fluctuating and migratory appearance over the course of several computed tomography (CT) scans and spontaneous resolution of lesions [4]. There are also specific perilobular abnormalities unique to OP that include arcade-like or curved bands of parenchymal consolidation with blurred borders. These are typically distributed along the structures that surround the secondary pulmonary lobule and often reach the pleura surface [4].

Although cases have described OP secondary to electronic cigarette use [6], and a history of smoking is common in patients with focal OP [7], OP directly attributed to ongoing cigarette smoking does not appear to have been described before. We present a patient with a history as well as radiographic and pathologic findings thought to be consistent with lung cancer who was ultimately diagnosed with OP suspected to be related to concurrent cigarette smoking.

This article was previously presented as an abstract via podium presentation at the 2022 CHEST Annual Scientific Meeting in Nashville, TN, on October 17, 2022.

## Case presentation

A 70-year-old woman with active tobacco use with a 40-pack-year smoking history presented to her primary care physician for evaluation of dyspnea, cough with scant hemoptysis, and an unintentional 11-pound weight loss over several months. She reported chills, night sweats, and pleuritic chest pain. The differential diagnosis initially favored malignancy; other pathologies considered included bacterial pneumonia, acute eosinophilic pneumonia, OP, vasculitis, interstitial lung disease, endemic mycoses, and pulmonary hemorrhage. She had a broad inflammatory and serologic evaluation with normal levels of the following tests: C-reactive protein, erythrocyte sedimentation rate, antinuclear antibody, anti-SSA/SSB, anti-Scl-70, anti-cyclic citrullinated peptide, rheumatoid factor, creatine kinase, aldolase, and components of the hypersensitivity pneumonitis panel. No clear causes of infection were noted, as evidenced by bronchoalveolar lavage specimens obtained from the lingula with negative alpha-galactomannan antigen, pneumocystis jirovecii pneumonia PCR, and bacterial, fungal, and acid-fast bacilli cultures.

**Figure 1:**
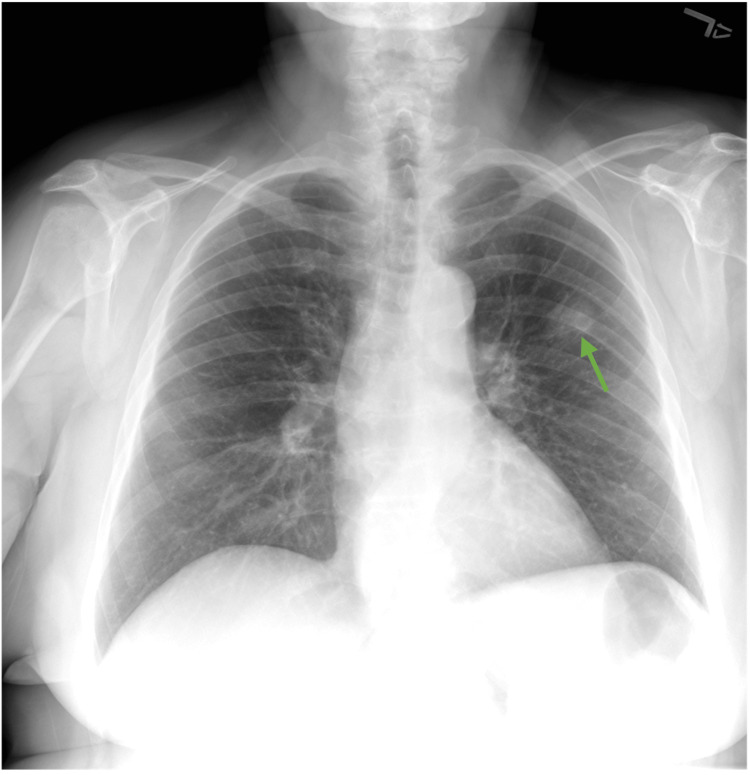
Initial PA chest radiograph revealing a 2 cm left upper lobe nodule (green arrow) PA: posteroanterior

Additionally, she tested negative for influenza, SARS-CoV-2, and respiratory syncytial virus during initial evaluation by her primary care provider. There were no changes in her smoking habits, recent infections, new medications or supplements, or pneumotoxic medications on the patient's medication list prior to the development of her symptoms. The CHEST Interstitial and Diffuse Lung Disease Patient Questionnaire did not reveal any clear-provoking etiology. However, the initial chest radiograph revealed a left upper lobe (LUL) nodule (Figure 1).

**Figure 2:**
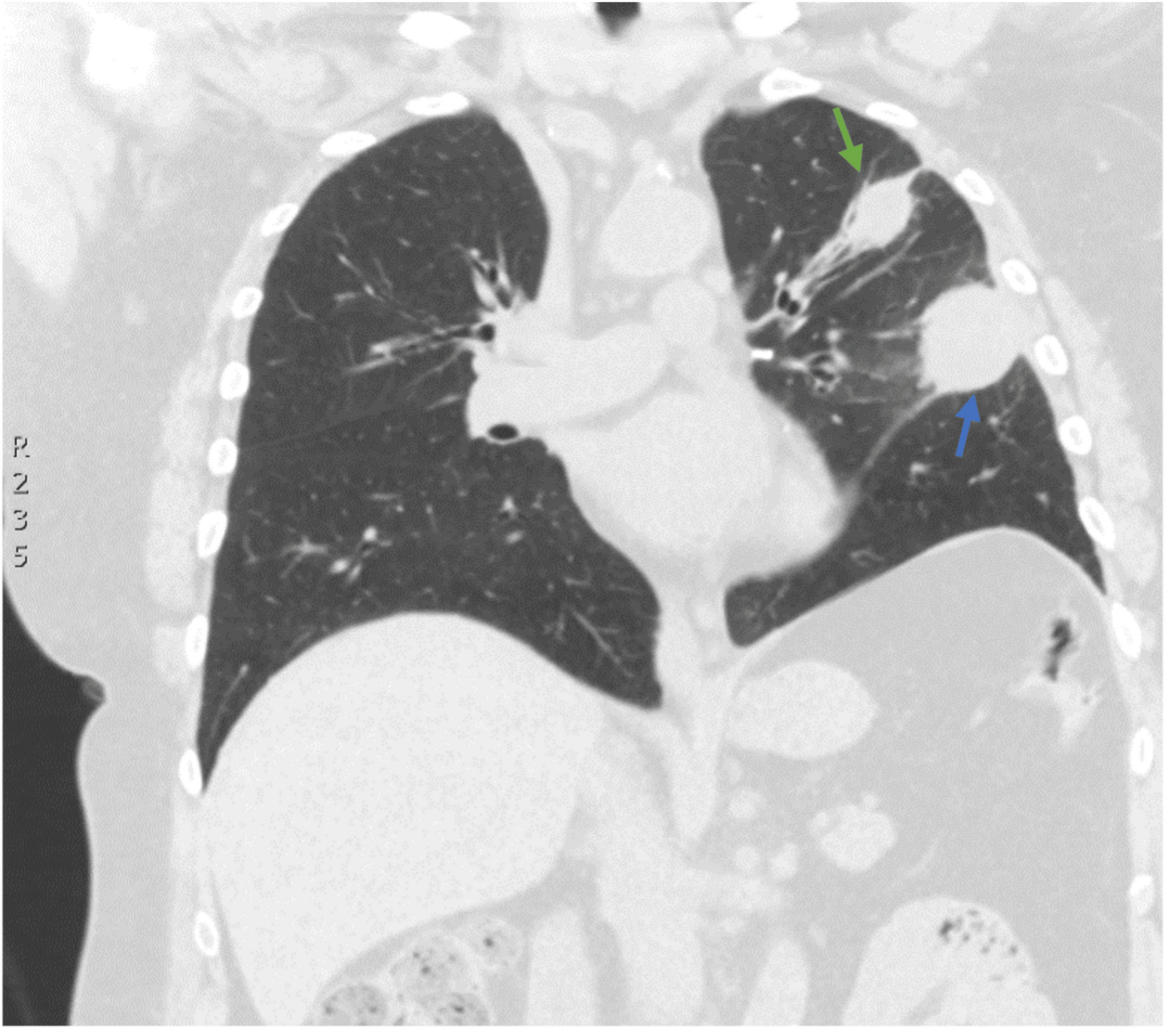
CT chest without contrast obtained during the early stages of the initial evaluation Coronal view demonstrating a mildly spiculated left upper lobe nodule, measuring 2.3x2x2 cm (green arrow), and a 4x3.5x3.5 cm mass invading the major fissure (blue arrow). Left-sided pleural thickening was also demonstrated. Slice thickness: 3 mm. Edge-enhanced reconstruction algorithm.

**Figure 3:**
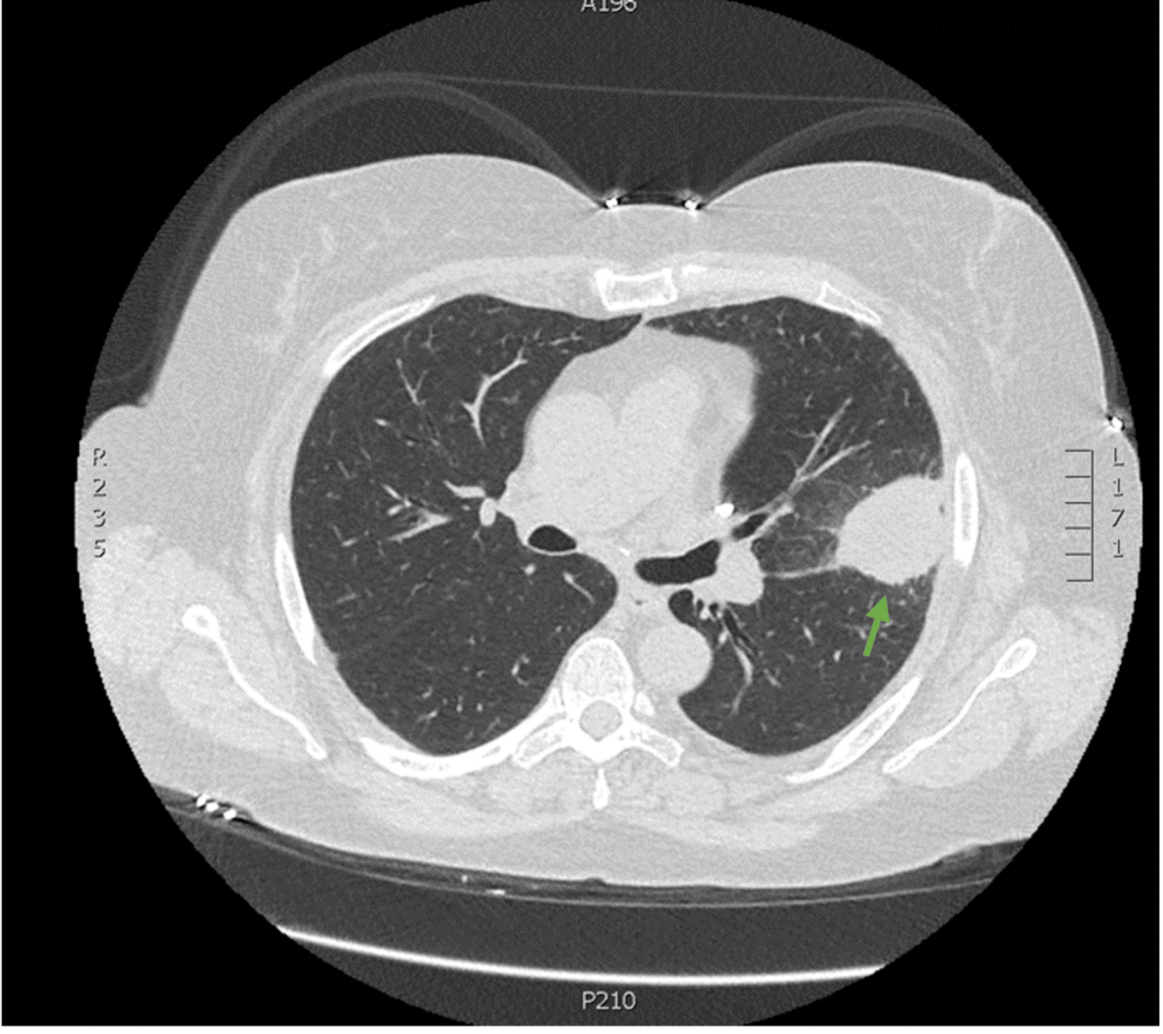
Axial view of the CT chest without contrast revealing a 4x3.5x3.5 cm mass invading the major fissure (green arrow) Slice thickness: 1 mm. Edge-enhanced reconstruction algorithm.

The CT chest revealed a 4 cm pleural-based mass in the LUL with invasion of the major fissure, a 2.3 cm spiculated nodule also in the LUL, a 2.1 cm left lower lobe spiculated nodule, and a left-sided pleural effusion, which was too small to sample (Figures 2, 3).

**Figure 4:**
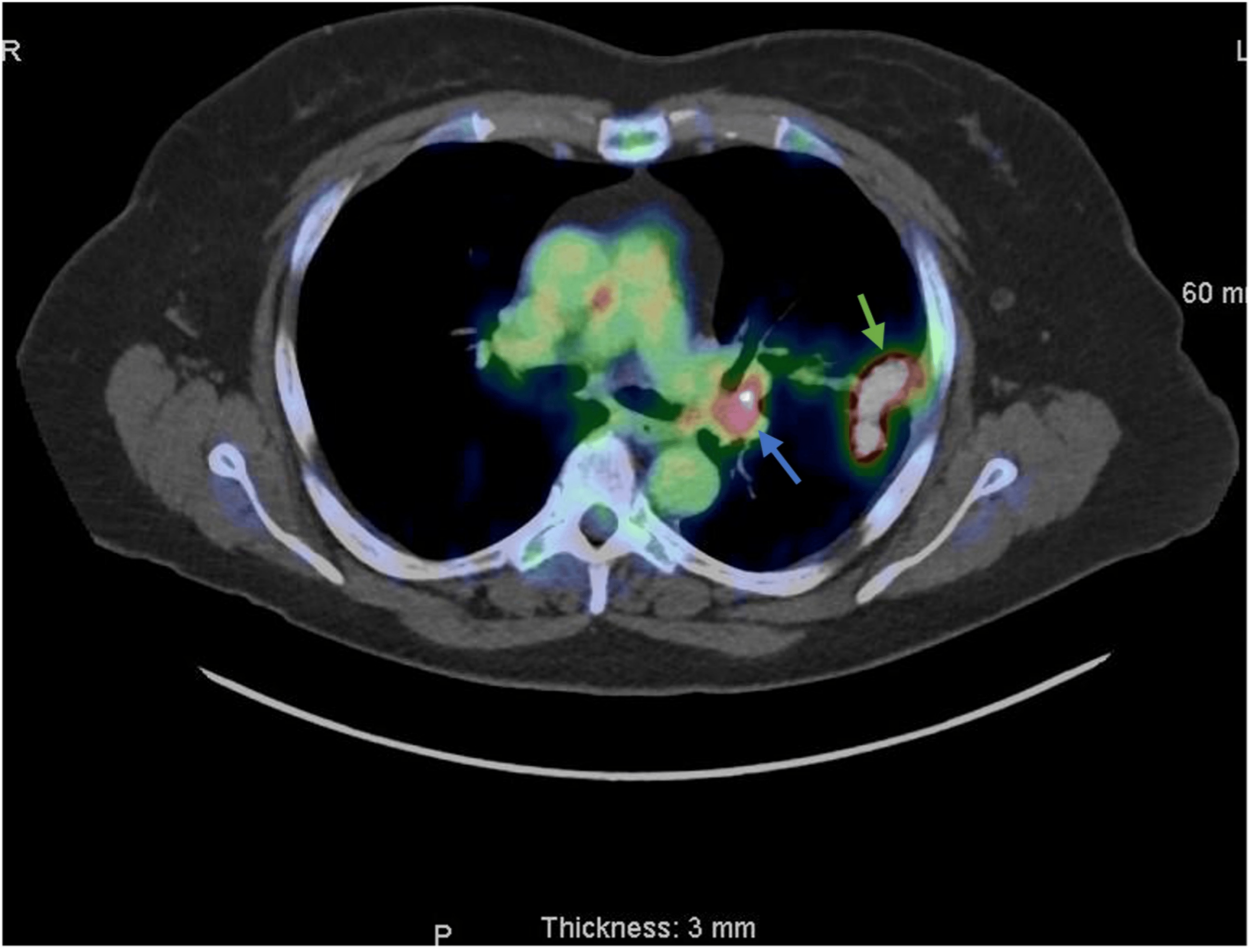
Axial view PET/CT revealing the FDG avidity of the left upper lobe (LUL) nodule (green arrow) and the ipsilateral mediastinal lymph nodes (blue arrow) The resolution of the pleural effusion and the decreasing size of the primary LUL nodule and mass are noted. Slice thickness: 3mm. Smooth reconstruction algorithm. PET/CT: positron emission tomography/computed tomography; FDG: fluorodeoxyglucose

One month after smoking cessation, positron emission tomography (PET)/CT revealed fluorodeoxyglucose (FDG) avidity but decreased size in the LUL mass and satellite nodule, alongside the resolution of the pleural effusion (Figure 4).

**Figure 5:**
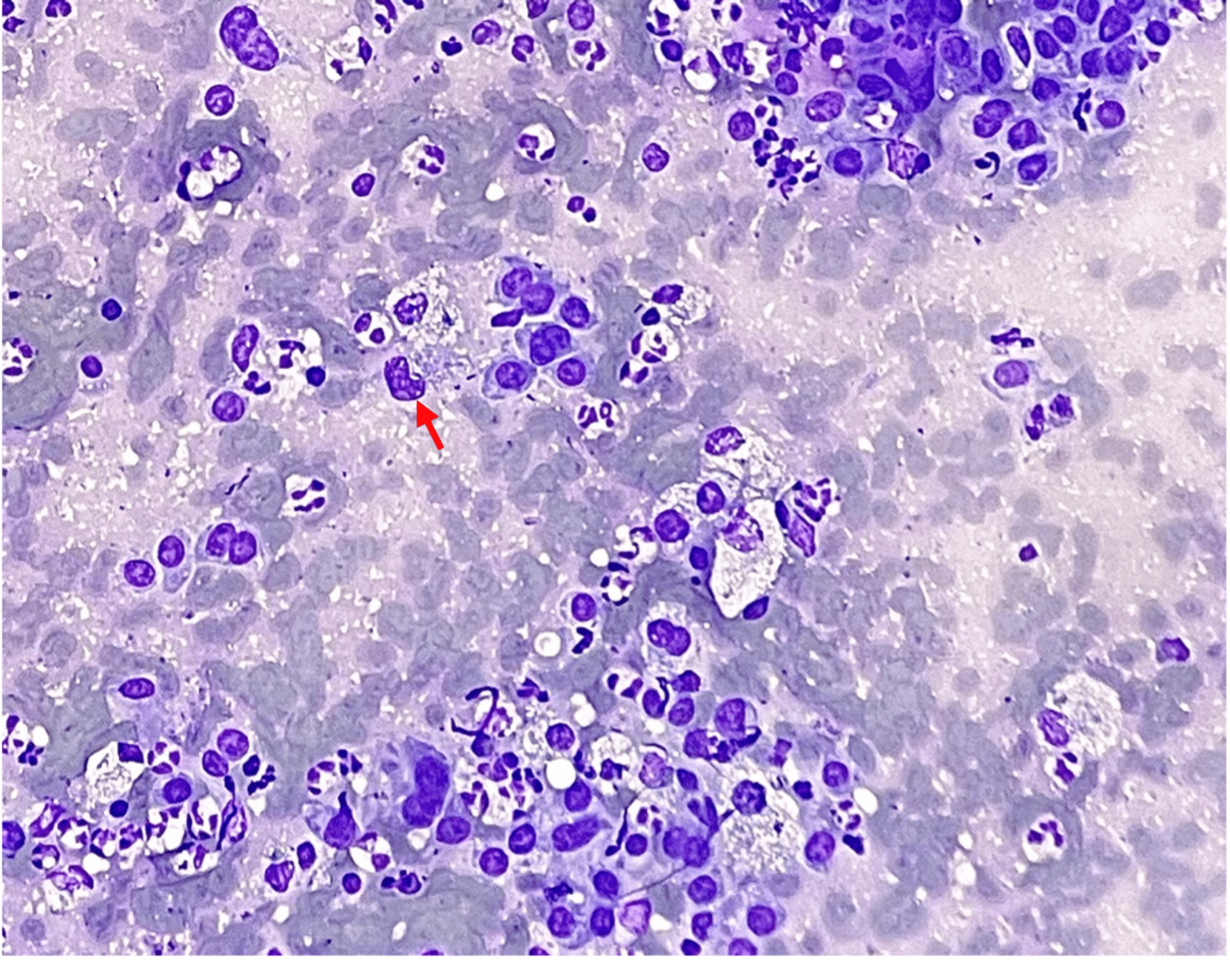
Left upper lobe nodule fine-needle aspirate obtained via navigational bronchoscopy Diff-Quik stain, 200×. Atypical cells with an increased nuclear-to-cytoplasmic ratio, irregular nuclear membranes, prominent nucleoli, and finely vacuolated cytoplasm (red arrow) are seen here. These cells present singly or in crowded clusters, with occasional anisonucleosis. Abundant acute inflammation is present in the background. On cell block, there is predominantly necroinflammatory debris with rare lesional cells present.

**Figure 6:**
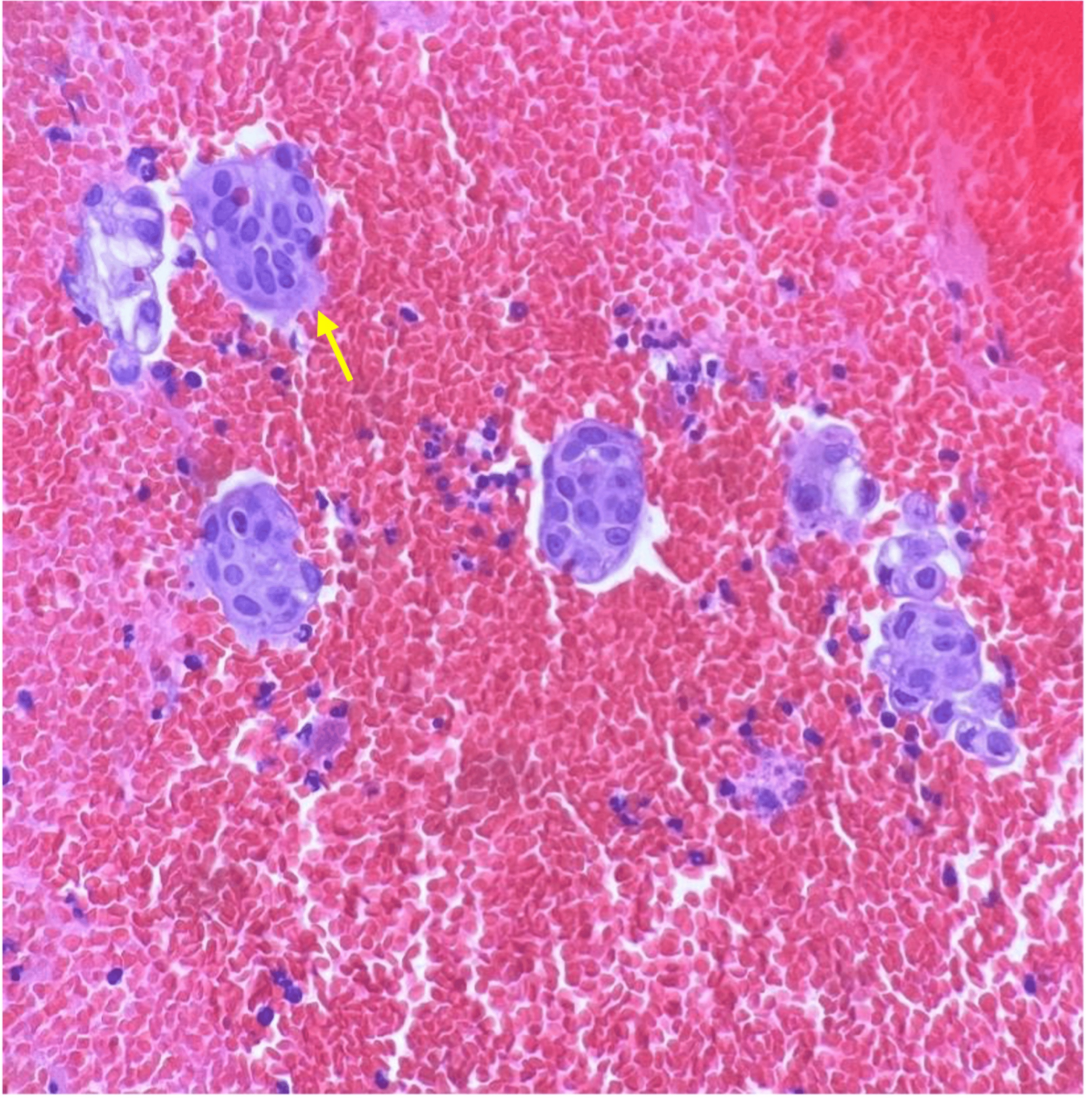
Left upper lobe nodule fine-needle aspirate obtained via navigational bronchoscopy H&E stain, 200×. Atypical cell blocks were noted with increased nuclear-to-cytoplasmic ratio, irregular nuclear membranes, prominent nucleoli, and finely vacuolated cytoplasm (yellow arrow). Rare lesional cells are p40 positive, with all cell blocks negative for TTF/Napsin. H&E: hematoxylin and eosin

Prior to one month of smoking cessation, an endobronchial ultrasound-guided fine-needle aspiration (FNA) of mediastinal lymph nodes was performed, along with navigational bronchoscopy with FNA of the LUL nodule. The LUL nodule was assumed to be a satellite nodule. Therefore, to obtain a higher TNM stage, it was biopsied initially via navigational bronchoscopy as the most likely site to yield diagnosis and stage. The pathology of the LUL satellite nodule FNA revealed rare lesional cells that were positive for p40 and negative for TTF-1/Napsin (Figures 5, 6).

**Figure 7:**
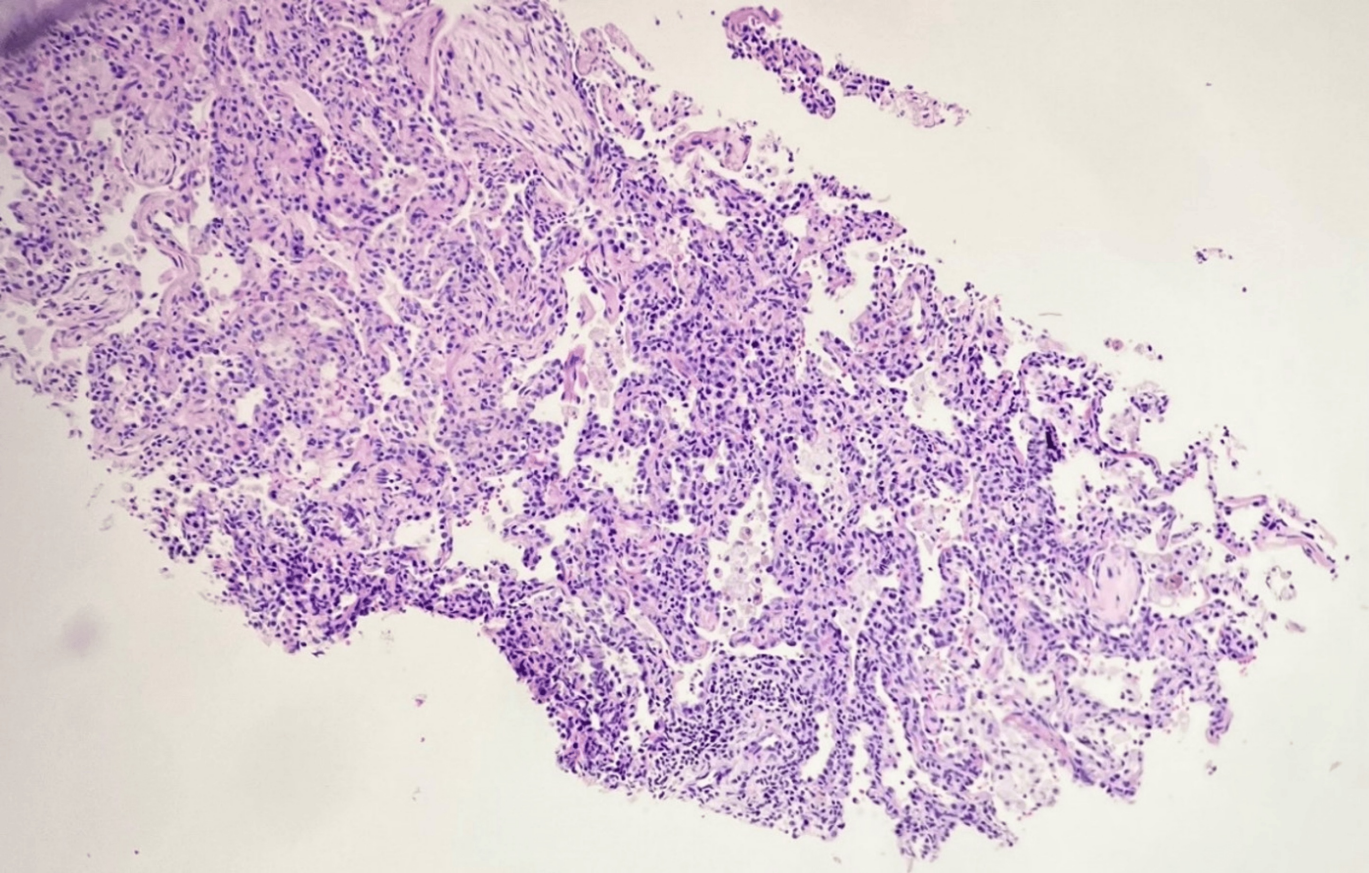
Low-power magnification of the left upper lobe mass core biopsy obtained by interventional radiology via CT guidance H&E (hematoxylin and eosin) stain, 100×.

**Figure 8:**
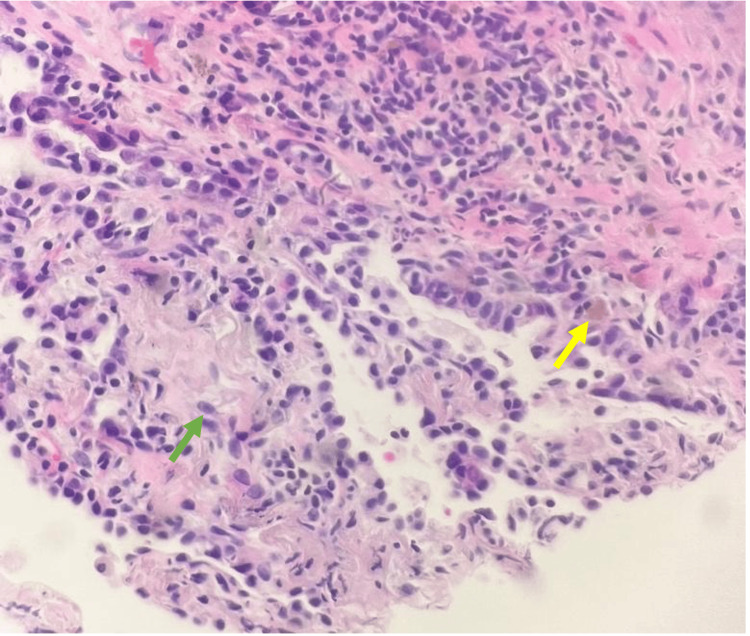
Left upper lobe lung biopsy cytologic touch preparations (200×, H&E stained) of the core biopsy This shows cells similar to those seen in the needle aspirate smears (green arrow). H&E stain. Sections of the lung show fibroblastic tissue arranged as buds in airspaces, filling and obliterating alveolar spaces and incorporating them into alveolar walls. This is associated with type II pneumocyte hyperplasia and minimal chronic inflammation. Neoplasm, granulomas, vasculitis, viral cytopathic effect, and aspirated food particles are not identified. The smoking-related injury of respiratory bronchiolitis (smokers' airspace pigmented macrophages) is present (yellow arrow). H&E: hematoxylin and eosin

**Figure 9:**
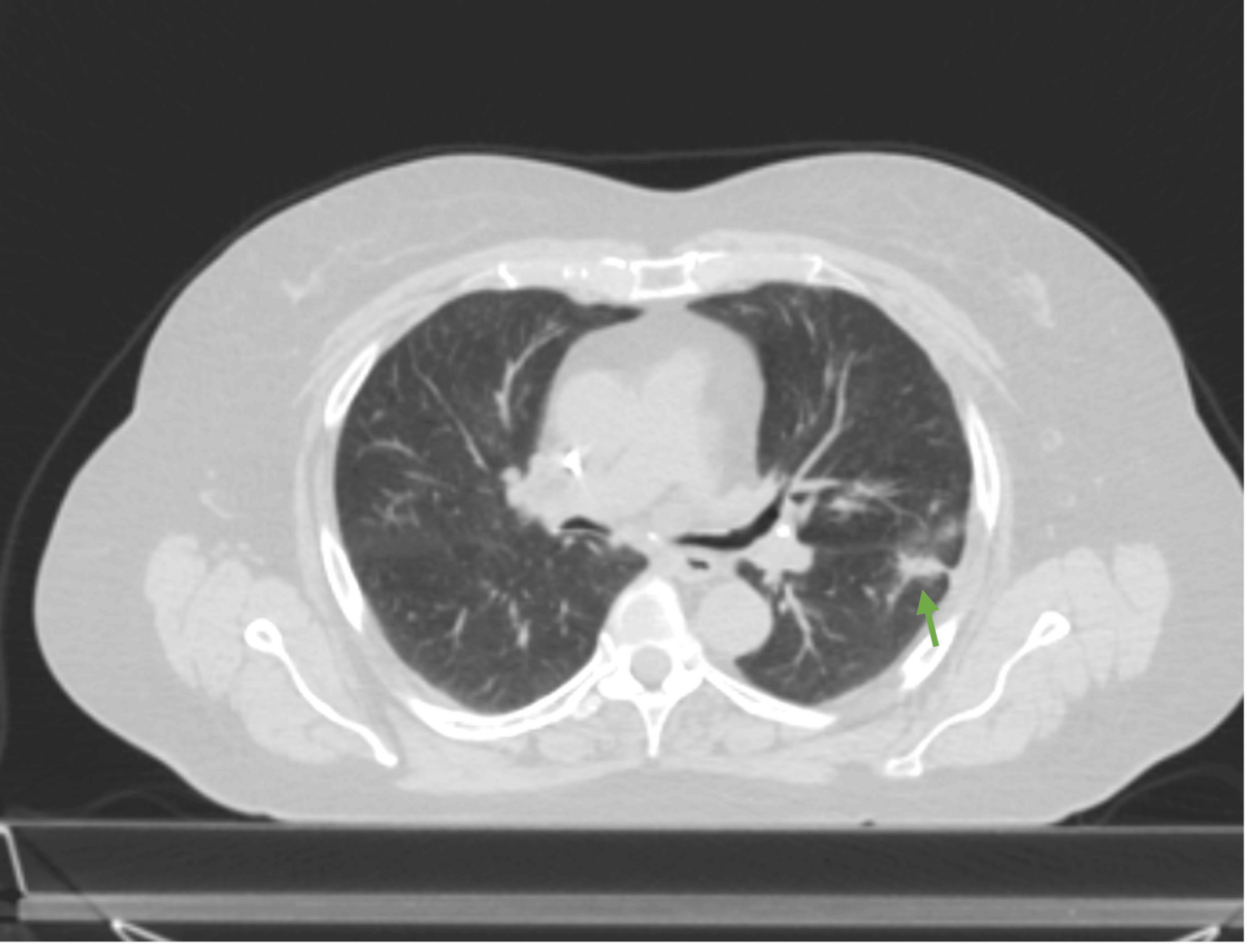
Axial view CT chest without contrast performed for radiation staging All nodules/masses are nearly resolved (green arrow). Slice thickness: 2mm. Edge-enhanced reconstruction algorithm.

The LUL nodule was reported as "neoplastic cells present." The 11L (ipsilateral hilar) lymph node was noted to have a rare focus of squamous cells with mild nuclear atypia that was positive for p40. Both cell blocks were noted to be in the background of abundant acute inflammation. The 4R, 7, 4L, and 10L lymph nodes were negative for carcinoma. A multidisciplinary tumor board met and discussed the patient's imaging and histopathology results. The patient was counseled on a likely diagnosis of T4N1M0 stage IIIA non-small-cell lung cancer, and she quit smoking. Plans were made to obtain more tissue from the dominant lesion for biomarker testing and to begin systemic chemotherapy. One month after smoking cessation, a CT-guided core biopsy of the LUL mass was performed and revealed thickened alveolar walls, chronic inflammation, and hemosiderin-laden macrophages (Figures 7, 8), findings consistent with OP with smoking-related inflammatory changes. Imaging during this biopsy showed that the LUL mass had significantly decreased in size and nearly resolved (Figure 9).

The tumor board diagnosis was changed based on this core biopsy of the dominant lesion, and the patient avoided chemotherapy. Bronchoscopic alveolar lavage was without signs of infection or acute eosinophilic pneumonia. Surveillance CT chest three months later demonstrated complete resolution of previously observed abnormalities.

## Discussion

Clinical discussion

OP is usually a diagnosis of exclusion. To the best of our knowledge, smoking as the direct cause of OP has not been reported in the literature. OP involves pulmonary injury followed by tissue repair that can be idiopathic (i.e., cryptogenic) or due to exposure to noxious stimuli [4]. Symptoms of COP, the idiopathic form of OP, were noted in the introduction [5].

Most cases of OP resolve after the removal of an offending agent or with the administration of high-dose corticosteroids and commonly relapse after treatment [5]. Our patient's symptoms and imaging abnormalities improved and resolved after one and three months of smoking cessation, respectively. The patient had a complete serologic workup for infectious and inflammatory causes of her pulmonary opacities. There were no new respiratory exposures, travel, medications, or recent infections found to have caused her findings. Given this time frame and our patient's otherwise negative infectious and inflammatory workup, her OP was attributed to tobacco use, given her improvement with smoking cessation. If she had continued to smoke and the same clinical outcome had occurred, we would be more inclined to label this as COP.

The clinical suspicion for lung cancer was high in this patient, given her symptoms in conjunction with her age, smoking history, and the nature of her pulmonary opacities (large, spiculated masses/nodules with surrounding ground glass opacities). An unusual aspect of her initial preliminary diagnosis of squamous cell carcinoma (SCC) was that her symptoms developed rapidly for what would have been a locally advanced lung cancer.

Radiologic discussion

The radiologic presentation of OP can be similar to that of lung cancer. Our patient presented with both a subpleural spiculated lung mass and two spiculated nodules with surrounding ground glass opacities that, even after a month of smoking abstinence, were PET avid. Pulmonary nodules with spiculated borders are highly suggestive of malignancy; however, they can also represent infectious or inflammatory processes [8]. The multifocal nature of the opacities with surrounding ground glass opacities and the subpleural location of the dominant lesion should have raised concern for the classic form of OP. The nodular form of OP can present with solitary or multiple nodules that may be spiculated and show FDG avidity [4]. Therefore, distinguishing between malignancy and inflammatory processes such as OP was difficult to do based on our patient's radiographic findings. However, the near and complete resolution of our patient's lung abnormalities at one and three months after smoking cessation makes lung cancer unlikely. Given this temporal relationship, smoking was assigned as the most likely cause of our patient's OP. Although we cannot rule out COP, COP does not resolve without steroids, treatment of an offending infection, or removal of the offending agent (in cases of secondary OP).

Some pathologic findings of our patient's lung disease suggested SCC. However, SCC is typically located centrally, and calcifications are common. Our patient's lung nodules were located peripherally, and no calcifications were evident, arguing against a diagnosis of SCC.

Pleural effusions are generally uncommon in COP [9]. The small pleural effusion noted on initial CT imaging was another atypical feature of our case and pushed us more toward a diagnosis of lung cancer. However, the effusion's resolution with smoking cessation gave us pause and necessitated the consideration of alternative etiologies, such as OP (Figure 9).

Pathologic discussion

The pathophysiologic progression of OP starts with the migration of inflammatory cells and plasma proteins into the alveolar space [10]. This is followed by fibroproliferative bud formation (i.e., organization). Afterwards, these fibroblasts and inflammatory cells disappear, leaving collagen behind. We believe that the timing of our patient's nodule and mass samplings resulted in obtaining different histopathologic stages of OP.

The diagnostic picture for our patient was clouded by histological staining that suggested lung cancer. Given that our patient's LUL nodule was negative for thyroid transcription factor-1 (TTF-1)/Napsin, which are reasonably sensitive and specific markers for adenocarcinoma of the lung [11], we placed adenocarcinoma lower on our differential diagnosis. The LUL nodule and 11L lymph node stained positive for p40, a highly sensitive and specific marker for SCC of the lung [12], and showed atypical squamous cells, suggesting a diagnosis of SCC. With the biopsy cell appearance and staining, our tumor board felt there was enough information to diagnose SCC, although recognizing that the background of chronic cellular inflammation was not typical. We suspect such atypical cells were present due to the biopsy obtained during the pathophysiologic process of OP. A specimen of low cellularity obtained from a highly inflammatory and cellular process like OP is the suspected reason for the malignant appearing cell population.

The patient was given a diagnosis of lung cancer at this point. Due to the paucity of cells obtained via FNA, our tumor board recommended additional tissue be obtained for biomarker testing with a core biopsy of the dominant LUL mass. Histologic testing of the LUL mass weeks later revealed fibroblastic tissue arranged as buds without signs of malignancy (Figures 7, 8). These findings are classic for OP.

Sampling of a satellite node will upstage the patient during a lung cancer evaluation if neoplasia is present. This was obtained initially in our patient via navigational bronchoscopy with FNA. We hypothesize that our patient was in the early stages of OP during the first biopsy, yielding these p40-positive inflammatory cells. It is suspected that this time difference allowed the development of the fibroproliferative buds that are characteristic of OP.

The diagnosis of SCC was based on seeing atypical cell blocks of p40 positivity in a satellite node with ipsilateral lymph node involvement obtained via FNA. Fortunately, a multidisciplinary tumor board recommended obtaining more tissue from the dominant mass via CT-guided core needle biopsy, which yielded more tissue and a clear histopathologic diagnosis of OP. The reversal of our patient's diagnosis highlights the importance of multidisciplinary tumor boards in the evaluation, diagnosis, and treatment of lung cancer.

## Conclusions

We discussed the case of a 70-year-old woman who was an active, longstanding smoker who presented with pulmonary and constitutional symptoms. She was found to have left-sided lung findings concerning for malignancy based on initial radiographic and histopathologic results. However, these findings resolved with smoking cessation and repeat tissue biopsy, which supported a diagnosis of smoking-related OP. This case demonstrates that the radiologic findings of OP may be mistaken for those of lung cancer, and it can be difficult even for skilled specialists to diagnose lung cancer based on histopathology. Multidisciplinary tumor boards and serial reevaluations of the disease may improve the ability of clinicians to diagnose and treat lung cancer and to distinguish malignancy from OP. The resolution of pulmonary lesions after smoking cessation supports a diagnosis of smoking-related OP. Patients with a clinical picture concerning for lung cancer should be encouraged to quit smoking promptly and early in the course of their workup, as improvement in radiographic findings and the evolution of histopathologic results may clarify the diagnosis. Further investigation is needed to elucidate the time course over which these changes can occur after smoking cessation in OP.
